# Utilization of multigene panels in hereditary cancer predisposition testing: analysis of more than 2,000 patients

**DOI:** 10.1038/gim.2014.40

**Published:** 2014-04-24

**Authors:** Holly LaDuca, A J Stuenkel, Jill S. Dolinsky, Steven Keiles, Stephany Tandy, Tina Pesaran, Elaine Chen, Chia-Ling Gau, Erika Palmaer, Kamelia Shoaepour, Divya Shah, Virginia Speare, Stephanie Gandomi, Elizabeth Chao

**Affiliations:** 1 Department of Clinical Diagnostics, Ambry Genetics, Aliso Viejo, California, USA; 2 Department of Genetic Counseling, Arcadia University, Glenside, Pennsylvania, USA; 3 Division of Genetics and Metabolism, Department of Pediatrics, University of California–Irvine, Irvine, California, USA

**Keywords:** hereditary cancer, moderate-penetrance genes, multigene panels, next-generation sequencing, targeted panels

## Abstract

**Purpose:**

The aim of this study was to determine the clinical and molecular characteristics of 2,079 patients who underwent hereditary cancer multigene panel testing.

**Methods:**

Panels included comprehensive analysis of 14–22 cancer susceptibility genes (*BRCA1* and *BRCA2* not included), depending on the panel ordered (BreastNext, OvaNext, ColoNext, or CancerNext). Next-generation sequencing and deletion/duplication analyses were performed for all genes except *EPCAM* (deletion/duplication analysis only). Clinical histories of ColoNext patients harboring mutations in genes with well-established diagnostic criteria were assessed to determine whether diagnostic/testing criteria were met.

**Results:**

Positive rates were defined as the proportion of patients with a pathogenic mutation/likely pathogenic variant(s) and were as follows: 7.4% for BreastNext, 7.2% for OvaNext, 9.2% for ColoNext, and 9.6% for CancerNext. Inconclusive results were found in 19.8% of BreastNext, 25.6% of OvaNext, 15.1% of ColoNext, and 23.5% of CancerNext tests. Based on information submitted by clinicians, 30% of ColoNext patients with mutations in genes with well-established diagnostic criteria did not meet corresponding criteria.

**Conclusion:**

Our data point to an important role for targeted multigene panels in diagnosing hereditary cancer predisposition, particularly for patients with clinical histories spanning several possible diagnoses and for patients with suspicious clinical histories not meeting diagnostic criteria for a specific hereditary cancer syndrome.

## Introduction

The cost of DNA sequencing has decreased significantly with the use of next-generation sequencing (NGS) technologies.^1^ Five years ago, NGS was used primarily in the research setting.^2^ Today, it is the primary sequencing method used for a variety of clinical genetic tests, including cell-free DNA for noninvasive prenatal testing, whole-exome sequencing, and a growing number of targeted multigene panels.^3,4^ Targeted panels have been used to aid in the diagnosis of a number of heterogeneous genetic conditions, such as cardiomyopathies, epilepsy, congenital muscular dystrophy, and X-linked intellectual disability.^5,6,7^ Results from recent studies have confirmed multiple advantages to the utilization of NGS panels in cancer genetic testing, including time and cost effectiveness as compared with the time and cost effectiveness of Sanger sequencing and deletion/duplication analyses of each gene separately.^8,9^ There is also an increased sensitivity or likelihood of detecting an affected individual's disease-causing mutation(s) due to the analysis of multiple genes simultaneously. An additional advantage is the potential for unexpected identification of mutation carriers for well-known cancer susceptibility syndromes in families with atypical phenotypes.^8,10^ Of note, Walsh et al.^10^ identified three *TP53* mutation carriers without a family history of Li–Fraumeni syndrome and two *MSH6* mutation carriers without a family history of Lynch syndrome in a study of 360 ovarian cancer patients unselected for age or family history.

Conditions with significant genetic heterogeneity for which NGS-based testing has been demonstrated as an effective testing method include hereditary breast and ovarian cancer and the hereditary colorectal cancer/polyposis syndromes.^8,9,10,11,12^ In March 2012, our laboratory began offering four clinical hereditary cancer panels. The purpose of this study is to report the clinical and molecular characteristics of 2,079 patients who underwent hereditary cancer multigene panel testing at our laboratory.

## Materials and Methods

### Participants

Study subjects included the first 2,079 patients who had hereditary cancer panel results reported by our laboratory between March 2012 and May 2013, prior to the inclusion of *BRCA1* and *BRCA2* on relevant panels (Ambry Genetics, Aliso Viejo, CA). All patients were clinician referred, and ordering standards were based on clinician judgment or clinic-specific thresholds. For the purposes of our retrospective data analysis, data were anonymized with the removal of all patient identifiers. This study was determined to be exempt from institutional review board review, and documented approval by the University of California–Irvine institutional review board for this exemption was obtained. Demographic and clinical history information—including gender, ethnicity, personal cancer history, family cancer history, and history of previous *BRCA1/BRCA2* testing—were collected from test requisition forms (TRFs) completed by ordering clinicians and submitted with patient specimens at the time of testing. In some instances, additional clinical information was obtained from clinic notes, pedigrees, previous test reports, and letters of medical necessity provided by the ordering clinician. Personal and family history information left blank on the TRF was interpreted as “not provided.” 

### Multigene panel design

An extensive research and development effort was undertaken to select genes for each multigene panel. Online databases (Human Gene Mutation Database and OMIM) and published literature were reviewed and manually curated, and genes were selected if evidence supported a minimum of a twofold increased risk for one of the cancers targeted by the respective panel (breast cancer for BreastNext; breast, ovarian, and uterine cancer for OvaNext; colorectal cancer for ColoNext; and breast, ovarian, uterine, and colorectal cancer for CancerNext) (**Supplementary Table S1** online). Herein, these panels will be referred to as breast panel, ovarian panel, colon panel, and cancer panel, respectively.

### Laboratory procedures

For all four hereditary cancer panels (breast panel, ovarian panel, colon panel, and cancer panel), NGS/Sanger sequencing was performed for all coding domains plus at least five bases into the 5′ and 3′ ends of all the introns and untranslated regions (5′UTR and 3′UTR) of 14–21 cancer susceptibility genes, depending on the panel ordered. Genes included on each panel are as follows: breast panel: *ATM*, *BARD1*, *BRIP1*, C*DH1*, *CHEK2*, *MRE11A*, *MUTYH*, *NBN*, *PALB2*, *PTEN*, *RAD50*, *RAD51C*, *STK11*, and *TP53*; ovarian panel: *ATM*, *BARD1*, *BRIP1*, *CDH1*, *CHEK2*, *EPCAM*, *MLH1*, *MRE11A*, *MSH2*, *MSH6*, *MUTYH*, *NBN*, *PALB2*, *PMS2*, *PTEN*, *RAD50*, *RAD51C*, *STK11*, and *TP53*; colon panel: *APC*, *BMPR1A*, *CDH1*, *CHEK2*, *EPCAM*, *MLH1*, *MSH2*, *MSH6*, *MUTYH*, *PMS2*, *PTEN*, *SMAD4*, *STK11*, and *TP53*; and cancer panel: *APC*, *ATM*, *BARD1*, *BRIP1*, *BMPR1A*, *CDH1*, *CHEK2*, *EPCAM*, *MLH1*, *MRE11A*, *MSH2*, *MSH6*, *MUTYH*, *NBN*, *PALB2*, *PMS2*, *PTEN*, *RAD50*, *RAD51C*, *SMAD4*, *STK11*, and *TP53*). For all ovarian, colon, and cancer panels, sequence analysis was not performed for *EPCAM* because currently the only mutations in *EPCAM* associated with Lynch syndrome are gross deletions encompassing the 3′ end of the gene.^13,14^ Genomic deoxyribonucleic acid (gDNA) was isolated from patients' whole-blood specimens using a QIAsymphony DNA kit (Qiagen, Valencia, CA). Saliva specimens were collected, and gDNA was isolated using an Oragene kit (DNAgenotek, Kanata, Canada). DNA was quantified using a spectrophotometer (Nanodrop; Thermoscientific, Pittsburgh, PA, or Infinite F200; Tecan, San Jose, CA). Sequence enrichment was carried out by incorporating the gDNA into microdroplets along with primer pairs designed to target hereditary cancer gene coding exons followed by polymerase chain reaction (RainDance Technologies, Billerica, MA). The enriched libraries were then applied to the solid surface flow cell for clonal amplification and sequencing using paired-end, 100-cycle chemistry on the Illumina HiSeq 2000 (Illumina, San Diego, CA). NGS analysis was then performed (Illumina). For all ovarian, colon, and cancer panels, *PMS2* sequence analysis was performed via Sanger sequencing due to pseudogene interference (*PMS2* is not included on the breast panel). For all panels, additional Sanger sequencing was performed for any region with insufficient depth of coverage for reliable heterozygous variant detection. Variant calls other than known nonpathogenic alterations were verified by Sanger sequencing in sense and antisense directions before reporting.

A targeted chromosomal microarray designed with increased probe density in regions of interest was used for the detection of gross deletions and duplications for each sample (Aglient, Santa Clara, CA). For all ovarian, colon, and cancer panels, *PMS2* deletion/duplication analysis was performed via multiplex ligation-dependent probe amplification due to pseudogene interference. If a deletion was detected in exons 12, 13, 14, or 15 of *PMS2*, double-stranded sequencing of the appropriate exon(s) of the pseudogene *PMS2CL* was performed to determine if the deletion was located in the *PMS2* gene or pseudogene.^15^

Initial data processing and base calling, including extraction of cluster intensities, was done using RTA 1.12.4 (HiSeq Control Software 1.4.5; Illumina). Sequence quality filtering was executed with the CASAVA software (version 1.8.2; Illumina, Hayward, CA). Sequence fragments were aligned to the reference human genome (GRCh37), and variant calls were generated using CASAVA. A minimum quality threshold of Q20 was applied, which translates to an accuracy of >99.9% for called bases; mean coverage was >300×.

Online databases including the Human Gene Mutation Database, the Single Nucleotide Polymorphism Database (dbSNP), 1000 Genomes, and HapMap, as well as online search engines (e.g., PubMed, OMIM, HGVS, and LOVD) were used to search for previously described variants.^16,17,18,19^ Variants were annotated with the Ambry Variant Analyzer, a proprietary alignment and variant annotation software (Ambry Genetics). Ambry's variant assessment program has developed a five-tier variant classification protocol based on published recommendations/guidelines by the American College of Medical Genetics and Genomics and the International Agency for Research on Cancer (**Supplementary Table S2** online).^20,21^ All variants, with the exception of previously characterized benign alterations, underwent thorough assessment and review of available evidence (e.g., population frequency information, published case reports, case/control and functional studies, internal co-occurrence and cosegregation data, evolutionary conservation, and in silico predictions) to arrive at a final variant classification. Results were reported as positive if one or more pathogenic mutations or likely pathogenic variants were detected, negative if no variants and/or only likely benign variants were detected, or inconclusive if only variants of uncertain significance were detected. In the event of a *MUTYH* mutation(s), only biallelic (homozygous/compound heterozygous) mutation carriers were considered to have positive results in this study, as *MUTYH*-associated polyposis is an autosomal recessive condition.^22^ Monoallelic (heterozygous) *MUTYH* mutation carriers were excluded from calculations. Calculated positive, inconclusive, and negative rates were based on current variant classifications at the time of manuscript submission. All variants, with the exception of benign alterations, were reported for all genes on the panel ordered. Detailed alteration and gene information was included on reports for any pathogenic mutations, likely pathogenic variants, and variants of unknown significance to support the reported classification, specific to the genetic alteration but not necessarily to the individual's clinical presentation. Thus, the ordering clinician retains responsibility for interpreting the test results in the context of a patient's clinical history.

### Data analysis

Retrospective TRF review was utilized for the collection of demographic and clinical history information and previous *BRCA1/BRCA2* test results. Reported personal and family cancer histories were manually reviewed and categorized as high-risk breast/ovarian if the appropriate criteria were satisfied (**Table 1**). Clinical histories for affected patients with positive results were reviewed to determine whether the reported clinical presentation of the patient or a confirmed carrier family member correlated with the gene-related cancer risk(s). For colon panel cases, in which mutations were identified in genes with well-established diagnostic criteria and treatment guidelines (with the exception of *CHEK2*), clinical histories were assessed to determine whether patients met clinical diagnostic/testing criteria.^23,24^

## Results

A total of 2,079 cases were included in our final data set: 874 breast panel, 557 colon panel, 223 ovarian panel, and 425 cancer panel samples. Demographic details for the 2,079 patients are provided in **Table 2**. The majority of patients (93.8%) had a personal history of cancer or adenomatous polyps reported by their health-care provider. Probands were reported as clinically unaffected in 4.8% of cases with a family history of cancer or adenomatous polyps. In the remainder of cases, clinical history information was not provided or other potentially significant history, such as hamartomatous polyps, was reported.

A total of 141 different pathogenic mutations were detected in 173 mutation-positive individuals (excluding monoallelic *MUTYH* mutations) (**Supplementary Table S3** online). The majority (*n* = 124; 87.9%) of identified mutations were nonrecurrent, and the remaining 12.1% (*n* = 17) were detected in more than one apparently unrelated individuals. Nearly all patients with positive results had a single pathogenic mutation detected (*n* = 160; 92.5%) or biallelic *MUTYH* mutations (*n* = 8; 4.6%), whereas five patients (2.9%) had two pathogenic mutations detected. Thirty-seven patients (21.4%) with positive results also had at least one variant of unknown significance detected.

**Figure 1:**
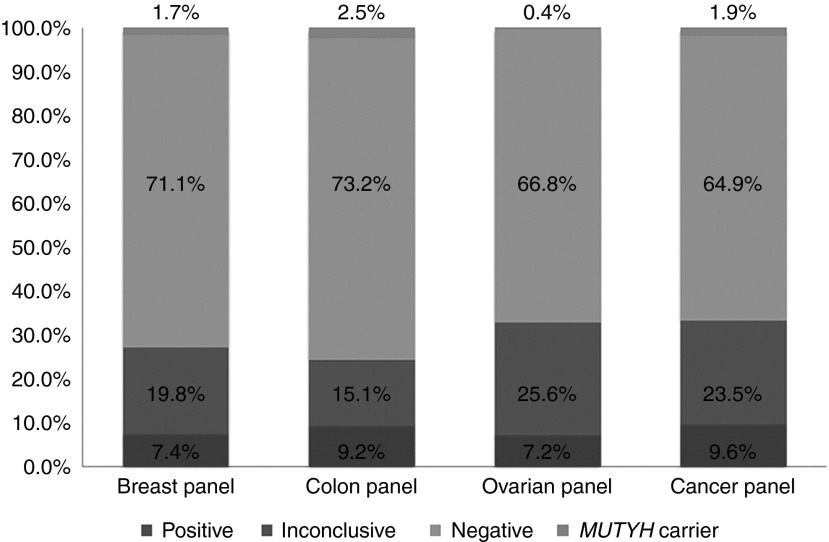
**Percentage of positive, inconclusive, and negative results by panel.**

Positive, inconclusive, and negative result rates varied by panel (**Figure 1**) and clinical characteristics (**Table 3**). The positive rate for the colon panel was comparable to those of the other panels; however, the inconclusive rate (15.1%) was the lowest, whereas the negative rate (73.2%) was the highest of the four panels. The positive rate for the ovarian panel was 7.2%; however, the inconclusive rate was 25.6%, which was the highest of all four panels.

Thirty-four patients in our cohort had a personal history of pancreatic cancer, four (11.8%) of whom received positive results. Three of these patients carried an *ATM* mutation, and one carried a *PALB2* mutation. Familial pancreatic cancer was reported for two of these four mutation carriers. The average age at pancreatic cancer diagnosis for mutation carriers was 53.8 years, which was earlier than the age for nonmutation carriers in this cohort (60.8 years).

### Correlation of results with gene-related risks

For affected patients with positive results (*n* = 64 for breast panel, 16 for ovarian panel, 50 for colon panel, and 40 for cancer panel cases), clinical histories correlated with gene-related risk(s) in 96.9% (*n* = 62), 75.0% (*n* = 12), 96.0% (*n* = 48), and 65.0% (*n* = 26) of breast, ovarian, colon, and cancer panel cases, respectively. For an additional 1.6% (*n* = 1) of breast, 18.8% (*n* = 3) of ovarian, 2.0% (*n* = 1) of colon, and 15.0% (*n* = 6) of cancer panel cases, clinical histories correlated with gene-related risks that have been suggested but not confirmed (e.g., ovarian cancer in an *MRE11A* mutation carrier, breast cancer in mismatch repair mutation carriers). For the remaining affected patients with positive results (*n* = 11 or 6.5% of total positive cases), clinical histories did not correlate with known gene-related cancer risks.

### Atypical phenotypes

Of the 557 colon panel patients who underwent testing, 51 (9.2%) were positive for a pathogenic mutation in at least one of the genes analyzed. Of these 51 cases with positive results, 46 (90.2%) carried pathogenic mutations in genes with well-established diagnostic criteria and treatment guidelines (*APC*, *CDH1*, *EPCAM*, *MLH1*, *MSH2*, *MSH6*, biallelic *MUTYH*, *PMS2*, *PTEN*, *SMAD4*, *STK11*, and *TP53*). The remaining five patients had mutations in *CHEK2*, which does not yet have set diagnostic criteria or guidelines. Of the 46 patients with pathogenic mutations in genes with well-established diagnostic criteria and treatment guidelines, 32 (69.6%) met the corresponding diagnostic criteria, and the remaining 14 (30.4%) did not.

## Discussion

The introduction of hereditary cancer multigene panels into the provision of clinical cancer services has raised concerns among genetics professionals. Panels are designed to include multiple genes, which may include high-penetrance genes as well as genes associated with a moderate increase in cancer risk(s). Limitations to a multigene panel testing approach have been addressed in the most recent update to the National Comprehensive Cancer Network Genetics/Familial High-Risk Assessment: Breast and Ovarian guideline,^23^ including the unknown level of risk for many genes, lack of clear guidelines for some genes, and unknown rates of variants of unknown significance. Recent publications have echoed several of these concerns and also emphasized the importance of developing new genetic counseling models for patients undergoing panel testing, as current models are not designed for this type of testing.^25,26^ In addition, there is limited information on potential risks and benefits of the multiplex approach from both the clinician and patient perspectives.

### Clinical interpretation of moderate-penetrance genes

Multigene panel testing is an effective strategy for identifying patients by genotype who meet a clinical management guideline. It is also an efficient resource for identifying eligible patients for screening and surveillance purposes, as well as for identifying qualified candidates for clinical patient registries. Arguably, the most common concern related to hereditary cancer panel testing is the clinical interpretation of findings in moderate-penetrance genes.^23,25^ Although clinical management guidelines or consensus opinions exist for the majority of genes included in the panels studied here (*APC*, *BMPR1A*, *CDH1*, *MLH1*, *MSH2*, *MSH6*, *MUTYH*, *PMS2*, *PTEN*, *SMAD4*, *STK11*, and *TP53*), management guidelines are not yet available for the moderate-penetrance genes (*ATM*, *BARD1*, *BRIP1*, *CHEK2*, *MRE11A*, *NBN*, *PALB2*, *RAD50*, and *RAD51C*). Mutations in these genes have been associated with a two- to fourfold increase in breast cancer risk and have each been associated with at least one other cancer type as well. In the absence of published management guidelines for individuals carrying mutations in these genes, clinicians are faced with the challenge of making medical management recommendations based on allele-specific risk information and individual patient clinical history.

Pathogenic mutations or likely pathogenic variants were not identified in the *BARD1* gene in this patient cohort. Although we have subsequently observed three *BARD1* mutation carriers in our cohort since the time of data collection for this study, the mutation frequency remains low. We will continue to review internal cosegregation, phenotype, and frequency data on *BARD1* mutations and variants in our cohort to aid in clarifying the contribution of this gene to hereditary cancer susceptibility.

### Correlation of results with gene-related risks

Genetic testing results correlated with reported clinical histories for the majority of affected probands with positive results in this study. The cancer panel resulted in the highest percentage (20.0%; *n* = 8) of probands whose genetic results did not correlate with known gene-related risks, in spite of this panel having the greatest number of genes with the broadest range of cancer risks. Correlations are based on cancer risks associated with these genes at this point in time; therefore, it is possible that additional associations will surface in the future as these genes undergo further investigation. For example, three cancer panel probands with *BRIP1* mutations had reported clinical histories of endometrial cancer/adenomatous colon polyps, melanoma, or adenocarcinoma of the small intestine, none of which have been correlated with *BRIP1* mutations at the time of writing. Further study of families such as these may help elucidate additional cancer associations.

### Inconclusive results

A major consideration when deciding between a targeted, single-gene approach to genetic testing versus testing using a multiplex gene panel is the increased chance of receiving inconclusive results, which presents challenges for both patients and clinicians. The overall inconclusive result rates for the colon, breast, cancer, and ovarian multigene panels over the first year of testing were 15.1, 19.8, 23.5, and 25.6%, respectively. These rates are based on current variant classifications and are lower than initial inconclusive rates prior to any variant reclassifications and the introduction of our current five-tiered classification scheme. Initial inconclusive rates were 23.7, 32.5, 40.7, and 43.3% for the colon, breast, cancer, and ovarian panels, respectively. The decrease in inconclusive rates can be explained by a combination of accumulating data and an updated classification scheme. Multigene panel inconclusive rates are related to both the number of base pairs sequenced and the data available to classify variants as pathogenic or benign. The initial inconclusive rates for many of the cancer susceptibility genes were relatively high, due in part to the lack of locus-specific databases and published literature for use in variant assessment. However, the rapid accumulation of data from familial testing and the availability of updated population frequency databases have resulted in improved variant classification and overall decreases in inconclusive result rates.^16,27^ As additional data and evidence sources become available for use in variant classification, we expect that the decreasing trend in inconclusive result rates will continue across all panels.

### Negative results

Although molecular results were positive for 8.3% of cases in our cohort, 69.9% had negative panel results. It is possible that some patients carried mutations outside the reportable range for the genes analyzed, but the chance of this is low because the analytical sensitivity is 99% or greater for described mutations in genes on these panels. With a negative test result, the possibility remains that a mutation(s) in a gene not included on the panel or yet to be identified in association with cancer contributed to the patient's clinical history. Therefore, cancer risk assessment remains important in the event of a negative result because additional cancer screening and risk reduction options may still be indicated for the patient based on clinical history.^23,24,28^ We appreciate the complexity of interpreting negative mutation-specific tests in family members of a carrier of a mutation in one of the moderately penetrant genes on these panels. Continued data collection through analysis of cosegregation and longitudinal study of mutation carriers through the work of the Evidence-Based Network for the Interpretation of Germline Mutant Alleles and other research groups will be necessary to guide risk assessment in this situation.^29^

Whole-exome sequencing is clinically available and may be a helpful option for selected cancer families seeking a molecular diagnosis. The use of whole-genome and whole-exome sequencing in hereditary cancer genetics has already shown promising results in the research setting and has allowed for the identification of *PALB2* and *ATM* as pancreatic cancer susceptibility genes.^30,31^ Whole-exome sequencing also led to the identification of *MAX* as a hereditary pheochromocytoma susceptibility gene, and *POLD1* and *POLE* as susceptibility genes in hereditary colorectal cancer.^32,33^

### Gene patents

Until recently, a significant issue surrounding multigene panel testing for cancer susceptibility was that *BRCA1/BRCA2* could not be included due to patents held by Myriad Genetics. In the recent Association for Molecular Pathology v. Myriad Genetics case, the Supreme Court of the United States ruled that naturally occurring DNA is not patent eligible merely because it has been isolated, as it is a product of nature,^34^ which has resulted in the expansion of hereditary cancer panels at multiple laboratories in the United States. In fact, the National Comprehensive Cancer Network guideline as of this writing states that panels are “intended for individuals who have tested negative for high penetrance genes (e.g., *BRCA1/2*) and for those whose family history is suggestive of more than one syndrome,”^23^ but the inclusion of *BRCA1* and *BRCA2* in hereditary cancer panels does not increase the cost, lending itself to a more comprehensive and cost-effective method for evaluating patients for hereditary cancer predisposition.

### Atypical phenotypes

Thirty percent (30%) of colon panel probands with mutations in well-defined genes did not meet clinical criteria for the associated syndrome and/or would not have met their insurance company's specific criteria for coverage of genetic testing for the given syndrome. In addition, the family history spanned several possible diagnoses for several of these cases. Clinical histories for the majority of these patients have been previously reported.^35^ Full pedigrees were available for 6 of the 14 colon panel probands who did not meet clinical criteria for the associated syndrome. For the remaining eight cases, our interpretation was based on the clinical history provided on the TRF, leaving the possibility that clinical criteria may have been met if the clinical history provided on the TRF or obtained by the clinicians was not complete. Despite this limitation, these results illustrate several advantages of multigene panel testing. Cases in which clinical diagnosis is dependent on specific pathology, such as juvenile polyps as a recommended criterion for *SMAD4* testing, could be overlooked if the patient's and family's records are not accurate. In addition, discrepancy in the reporting of polyp histology among pathologists is well recognized and may hamper the ability of clinicians to target gene testing.^24,36^ Furthermore, in patients with Lynch syndrome, microsatellite instability and immunohistochemical staining results are not always indicative of mismatch repair defects.^37,38^ Although general criteria for genetic testing for a specific condition provide useful guidelines, these results demonstrate that syndrome-specific targeted genetic testing will result in missed diagnoses. Multigene panel testing allows clinicians to consider a broader range of phenotypes in a cost-effective and timely manner.

### Study limitations

One limitation of this study is the inherent selection bias of the cohort toward patients with clinical histories suggestive of hereditary cancer predisposition. Therefore, our reported mutation detection frequencies are applicable to high-risk populations but may not translate to the general cancer population. Due to the selection bias of our cohort, results from this study could not be used to provide gene-specific penetrance information or assess for novel cancer associations with these genes. Currently, efforts are under way by Ambry Genetics to facilitate cosegregation analyses and obtain pedigrees for mutation-positive patients/families. Clinical information requested on the TRFs is currently limited to affected family members, and in order to fully assess specific cancer risks, a complete pedigree would be required. Further pedigree analysis combined with cosegregation data will provide additional information on penetrance of some of the genes.

Another limitation of this study is that the majority of clinical history information collected was limited to clinician report and not based on direct medical record review. It is possible that the information provided was limited to what clinicians believed to be relevant and did not include all cancers in the family.

### Conclusions

Results from this study indicate that multigene cancer panels may play an important role in the diagnosis of hereditary cancer predisposition. In the case of atypical phenotypes, cancer risks may be recognized only after genotype data are available; these risks would be missed if testing were guided strictly by established single-gene/syndrome testing guidelines. Current National Comprehensive Cancer Network guidelines specify that multigene panels should only be ordered in consultation with a cancer genetics professional.^23^ Careful interpretation of results by individuals adequately trained to perform genetic counseling and assess cancer risk is critical for all patients undergoing hereditary cancer panel testing but is particularly important for the clinical interpretation of mutations in moderate-penetrance susceptibility genes and risk assessment for patients with negative results. Results from this study provide an initial framework for clinical research on benefits and limitations of multigene panel testing and management of patients with mutations in moderate-penetrance genes. These data also provide support to the clinician considering the multigene approach to genetic testing for hereditary cancer predisposition.

## Disclosure

H.L., A.J.S., J.S.D., S.K., S.T., T.P., E.C., C.-L.G., E.P., K.S., V.S., and S.G. are full-time employees of Ambry Genetics' commercial laboratory. E.C. is a paid consultant of Ambry Genetics. D.S. declares no conflict of interest.

## Figures and Tables

**Table 1:**
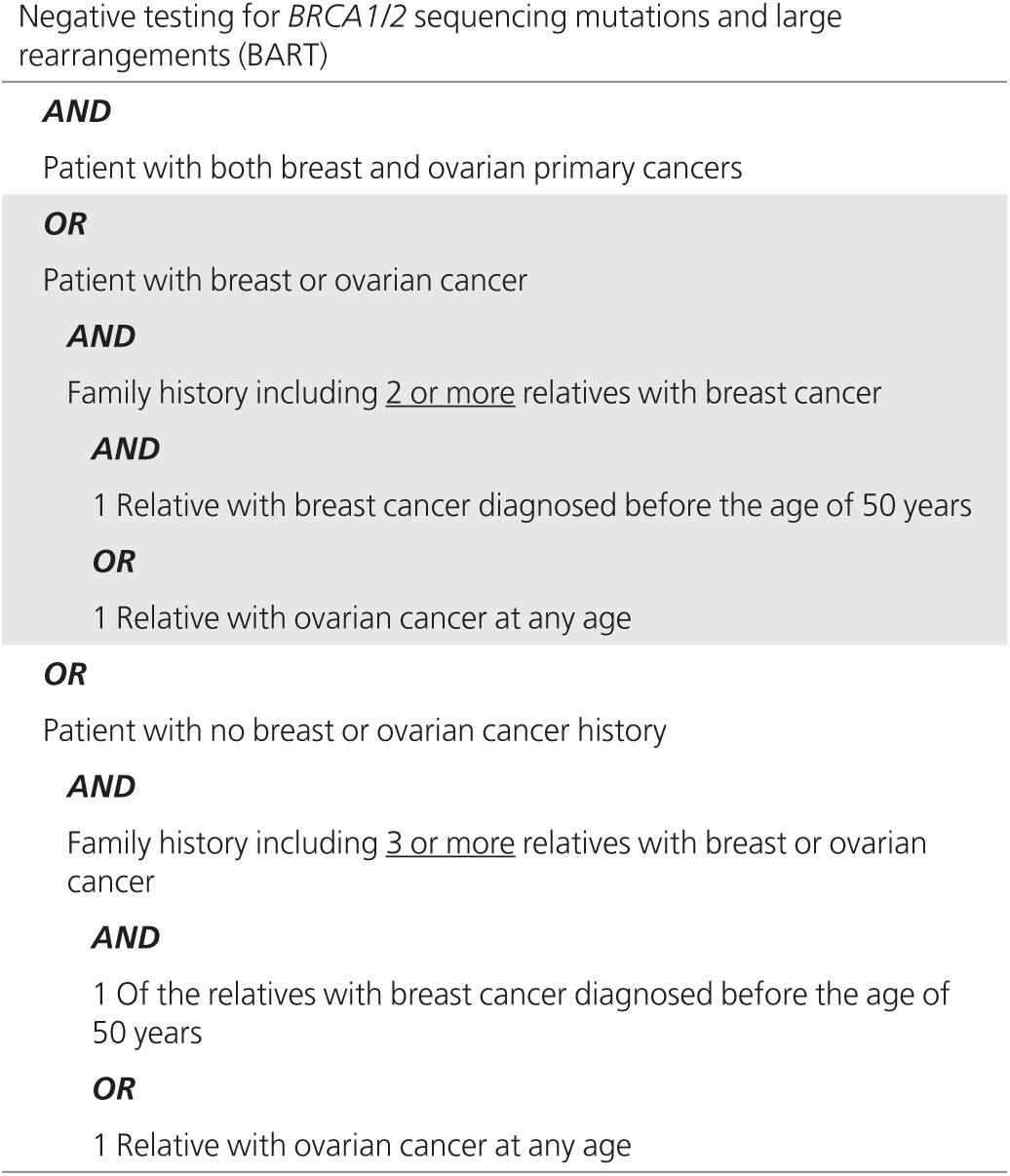
High-risk breast/ovarian cancer criteria

**Table 2:**
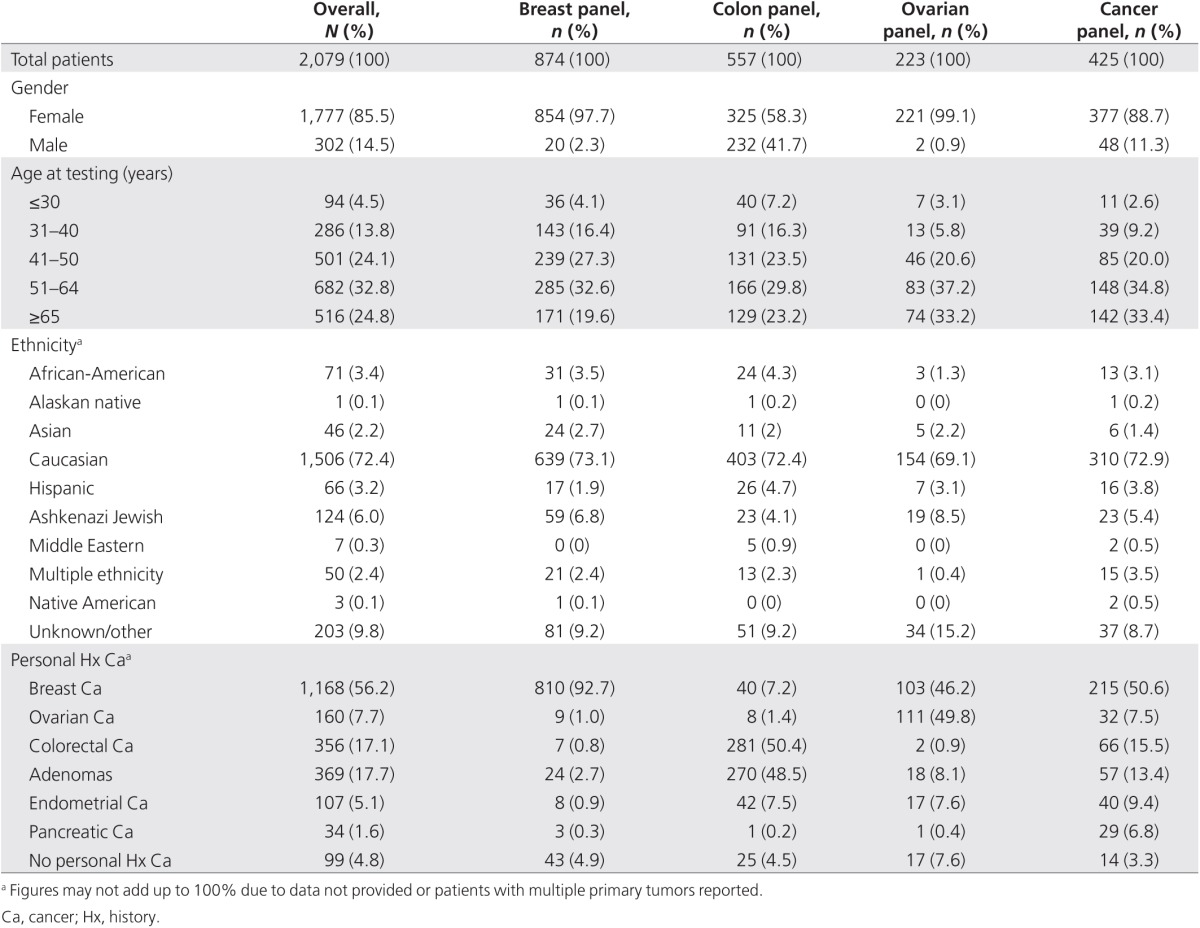
Patient demographics

**Table 3:**
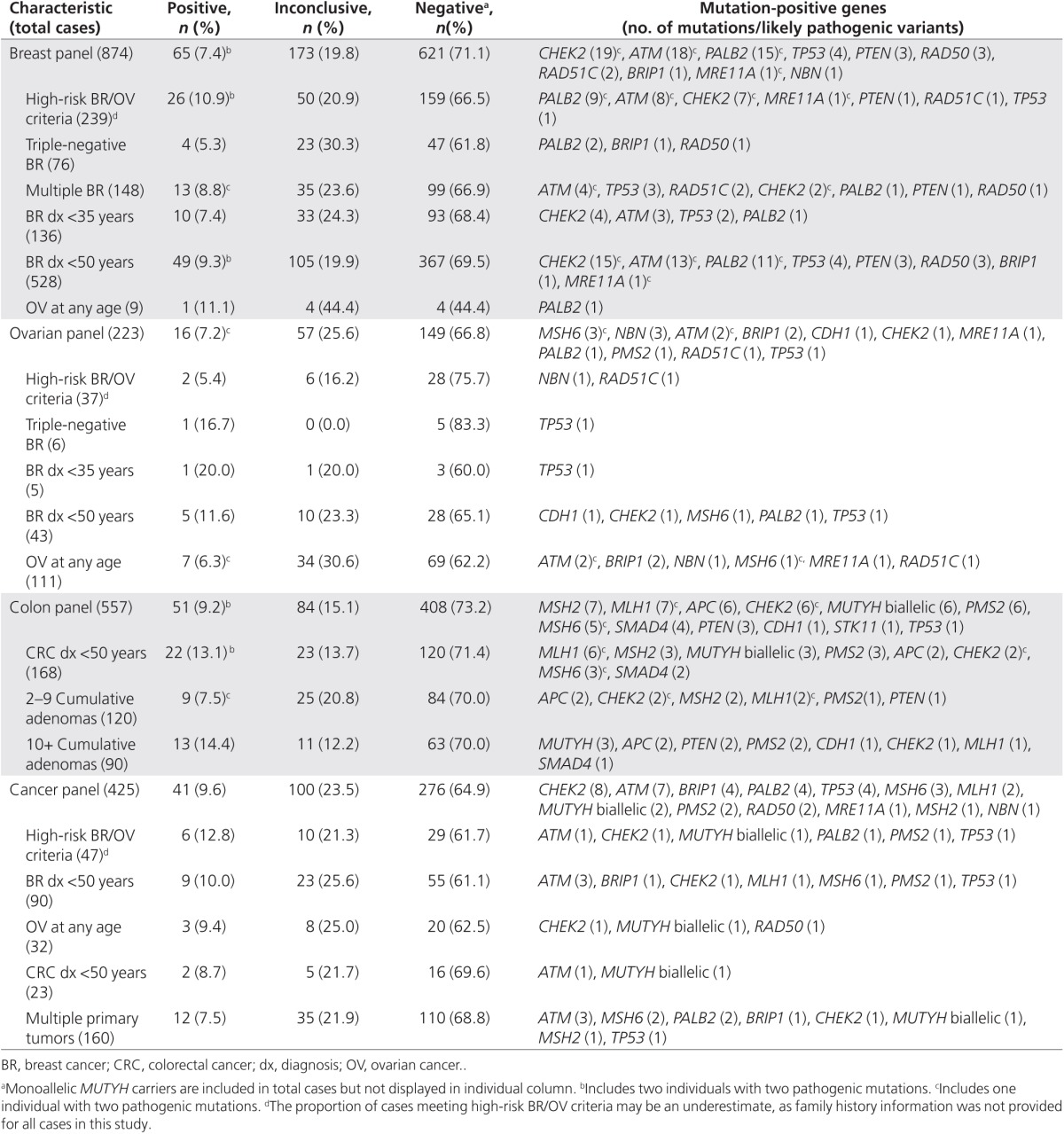
Result rates by panel and clinician-reported clinical history
